# Functional probiotics of lactic acid bacteria from Hu sheep milk

**DOI:** 10.1186/s12866-020-01920-6

**Published:** 2020-07-28

**Authors:** Taohong Chen, Leli Wang, Qinxin Li, Yingjie Long, Yuming Lin, Jie Yin, Yan Zeng, Le Huang, Tingyu Yao, Muhammad Nazeer Abbasi, Huansheng Yang, Qiye Wang, Congjia Tang, Tahir Ali Khan, Qiuyue Liu, Jia Yin, Qiang Tu, Yulong Yin

**Affiliations:** 1 Hunan Provincial Key Laboratory of Animal Intestinal Function and Regulation, College of Life Sciences, Hunan Normal University, Changsha, China; 2 Hunan International Joint Laboratory of Animal Intestinal Ecology and Health, College of Life Science, Hunan Normal University, Changsha, 410081 China; 3 Helmholtz International Lab for Anti-Infectives, Shandong University-Helmholtz Institute of Biotechnology, State Key Laboratory of Microbial Technology, Shandong University, Qingdao, 266237 China; 4 Institute of Genetics and Developmental Biology, the Innovation Academy for Seed Design, Chinese Academy of Sciences, Beijing, China; 5 Chinese Academy of Science, Institute of Subtropical Agriculture, Research Center for Healthy Breeding of Livestock and Poultry, Hunan Engineering and Research Center of Animal and Poultry Science and Key Laboratory for Agroecological Processes in Subtropical Regions, Ministry of Agriculture, Changsha, China

**Keywords:** Hu sheep milk, Probiotics, Antimicrobial activity, Cell surface characteristics, *Lactococcus lactis*, *Leuconostoc lactis*

## Abstract

**Background:**

Probiotics are being considered as valuable microorganisms related to human health. Hu sheep is referred as one of the important sheep breeds in China. Goat milk produced by Hu sheep is characterized with high nutritional value and hypoallergenic in nature. Particularly, this milk contains plenty of milk prebiotic and probiotic bacteria. This study was aimed to scrutinize more bacterial strains from Hu sheep milk with potential probiotic activity.

**Results:**

Based on 16S rRNA sequence analysis, pool of forty bacterial strains were identified and evaluated their antimicrobial activities against *Staphylococcus aureus*, enterohemorrhagic *Escherichia coli* (EHEC), *Salmonella typhimurium*, *Listeria monocytogenes* enterotoxigenic *E. coli* (ETEC) and *Aeromonas caviae*. Four out of these isolated strains demonstrated their efficient bacteriostatic ability and potential healthy properties. We also examined the safety aspects of these bacterial candidates including three *Lactococcus lactis* strains (named as HSM-1, HSM-10, and HSM-18) and one *Leuconostoc lactis* strain (HSM-14), and were further evaluated via in vitro tests, including antimicrobial activity, cell surface characteristics (hydrophobicity, co-aggregation, and self-aggregation), heat treatment, antibiotic susceptibility, simulated transport tolerance in the gastrointestinal tract, and acid/bile tolerance. The obtained results revealed that HSM-1, HSM-10, HSM-14, and HSM-18 showed high survival rate at different conditions for example low pH, presence of bovine bile and demonstrated high hydrophobicity. Moreover, HSM-14 had an advantage over other strains in terms of gastrointestinal tract tolerance, antimicrobial activities against pathogens, and these results were significantly better than other bacterial candidates.

**Conclusion:**

Hu sheep milk as a source of exploration of potential lactic acid bacteria (LAB) probiotics open the new horizon of probiotics usage from unconventional milk sources. The selected LAB strains are excellent probiotic candidates which can be used for animal husbandry in the future. Rationale of the study was to utilize Hu sheep milk as a source of potential probiotic LABs. The study has contributed to the establishment of a complete bacterial resource pool by exploring the Hu sheep milk microflora.

## Background

Previous definitions of probiotics emphasized its role in improving gut’s microbial ecosystem, suggesting that probiotics are vitally beneficial for the sustainability of intestinal tract and boast immune system [1]. Food and Agriculture Organizations and the World Health Organization, defines probiotics as live microorganisms that confers health benefits on their hosts when ingested in an adequate concentration [2]. In past decades antibiotics abuse has been reported to accelerate drug resistance and evolution of antibiotic resistant strains. Probiotic therapy can be an ideal alternative approach for treating various diseases without getting in the worry of drug resistance. Consumers increasingly needs natural probiotic foods to improve their health and well-being [3]. Lactic acid bacterial strains (LABs) are the members of the our intestinal microbiota and widely being used as probiotics [4]. The most commonly used probiotic *Lactobacillus* is a member of the normal intestinal microbiota and considered as Generally Recognized As Safe (GRAS) [5]. LABs are widely being used for the elaboration of fermented foodstuffs and increasingly being added to a growing number of foodstuffs such as cheese, yogurt, cereals, fruit, and vegetable juices [6]. LABs form microbial communities and a biological barrier which positively impacted on diarrhea, food allergies, and inflammatory bowel disease [7, 8]. *Lactococcus lactis* is one of the oldest domesticated bacterial species and commonly used to make nutritious and healthy foods throughout the world. Due to the current ban of the addition of antibiotics in feeds in China and furtherance of antibiotics free feeds, it has also been modified as an expression host for antimicrobial peptides and proteins, such as many kinds of antibiotics can be modified into a carrier or displayer of oral vaccines. The genus *Leuconostoc* is Gram-positive, catalase-negative and facultative anaerobes [9]. They can be present in natural food and can also be used as food additives because of their nutritional properties and organoleptic characteristics [10]. Studies have shown that the sensory characteristic properties of goat yogurt can be greatly improved by integrating the cultures of *Leuconostoc lactis* with traditional yogurt starters [11]. Moreover, *Leuconostoc lactis* is one of the most important species in the genus *Leuconostoc*, which plays an important role in the production of glucan and bacteriocin in the food industry [12, 13].

The gut microbiota forms a dynamic and diverse community, and certain lactobacilli have been proven to exert health benefits [14]. And the host’s treatment of intestinal diseases is also related to probiotics that improve the balance of intestinal flora. For example, treatment of 8-week-old Swiss mice fed a high-fat diet with a mixture of probiotics containing *Lactobacillus* could significantly change the composition of its intestinal flora and increase insulin sensitivity [15]. Similar studies on obesity indicated that microscopic fungi significantly reduced in the microbiota composition of obese mice treated with *Lactobacillus casei* and *Lactobacillus delbrueckii* subsp. *bulgaricus* [16].

In the cause of being applied as probiotic, each LAB candidate should be probed to ensure the safety and desirability of the strain [17]. During the process of bacterial colonization, first step is the bacterial adhesion to host’s tissues, which has always been long expected to be a compelling property among the probiotics [18]. The gastrointestinal tract of animals is considered as the most complex microbial ecosystems, and can affect the absorption and metabolism of nutrients, nutritional and protective functions of the host [19]. Meanwhile, as the normal inhabitants of the healthy gut microbiota, LABs can survive, colonize, and adhere to host tissues. In order to survive, probiotic has to adhere to the intestinal tract and tolerate the presence of bile salts and low intestinal pH. It would also be necessary to check the effect of gastric acid upon reduction of viability of probiotic cells. In addition, spray drying is an economical storage technology being used for probiotics processing, which offers dry and stable powdered form of probiotics and thought to be convenient in transportation which is widely used in dairy industry [20, 21]. However, heat-sensitive cultures have a low survival rate during spray drying procedures [21, 22]. Therefore, it is significant to determine the thermal stability of probiotics and to optimize thermal threshold, which could be cost effective during industrial usage. The heat-resistant strain has high survivability ratio and offers a series of industrial advantages [23].

Microbes in milk have been widely reported, but the majority of probiotics in milk samples were also found in human milk or bovine milk [24]. These microorganisms derived from breast milk have the advantage of direct vertical transmission which deserves more in-depth study. Sheep milk have high digestibility, high nutritional quality, low allergenicity, and potential nutraceutical properties [25]. Moreover, sheep milk also provides prebiotic fibers and probiotic bacteria [26, 27]. Hu sheep is a unique local sheep breed in Taihu Plain of China, and among the several white breeds in the world, which has long estrus period, good lactation performance and an average litter size of 2.06 [28], Due to several economic advantages for example fast growth, high productivity rate, good quality meat production, resistance to rough feeding and full house feeding Hu sheep bread is considered to be the first choice for factory producers in China [29]. Gut microorganisms are wildly reported to have a great influence on the growth performance of animals, and maternal milk microbes are the first colonizers in gut of mammalian offspring, which microbes were existed in Hu sheep milk? The aim of the present study was to evaluate potential traits of probiotic strains of three *Lactococcus lactis* strains (HSM-1, HSM-10, and HSM-18) and one *Leuconostoc lactis* strain (HSM-14) isolated from Hu sheep milk through using a series of in vitro tests*.*

## Results

### Screening and identification

**Fig. 1:**
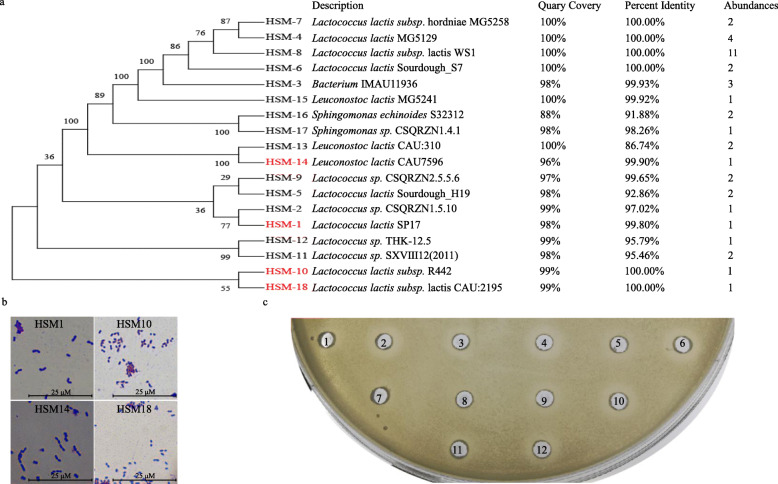
**a** The evolutionary history was inferred using the Maximum Parsimony method. The bootstrap consensus tree inferred from 500 replicates is taken to represent the evolutionary history of the taxa analyzed. Branches corresponding to partitions reproduced in less than 50% bootstrap replicates are collapsed. The percentage of replicate trees in which the associated taxa clustered together in the bootstrap test (500 replicates) are shown next to the branches whereas the BLAST alignment results are showed. The abundances of isolated bacterial are shown in the last column. **b** The microscope micrographs of four LAB strains. **c** Inhibitory effects of HSM-14 against the pathogen ETEC. The circle wells were filled in different products of HSM-14, including, fermentation broth of cells (pH 3.50) (1 &2) and cell-free supernatant (pH 3.50) without any treatment (3&4), with 15 min heat inactivation (5&6), with pH adjusting from 3.5 to 7.5 using NaOH (7&8), with the treatment of 1 mg/mL trypsin (9&10), and with the treatment of 1 mg/mL protease K (11&12). Some substances (1, 3, 5, 7, 9 and 11) were obtained from the fermentation broth after cultivation under the aerobic condition while others (2, 4, 6, 8, 10 and 12) were from the culture under anaerobic condition

After cultivation of microbes from Hu sheep milk, we obtained 40 isolates on the basis of their colony morphology and 16S rRNA gene sequences to cover as much diversity as possible at the species level. A phylogenetic tree (Fig. 1a) was constructed using the maximum parsimony analysis with MEGA-X and indicated the relative phylogeny of forty isolates from Hu sheep milk in comparison to reference strains [30, 31]. *Lactococcus lactis* were the most abundant specie in obtained microflora (30/40), reflecting the dominance of these species in Hu sheep milk. Minor populations of the other genera such as *Leuconostoc lactis* (4/40) and *Sphingomonas* (3/40) were also present in the phylogenetic tree. For further verification, the microscopic pictures of strains were examined (Fig. 1b). In the antimicrobial assay, the four selected strains were used for further delineation as they showed eminent probiotic candidates properties with highest antimicrobial activity against enterohemorrhagic *Escherichia coli* (EHEC)*,*enterotoxigenic *Esherichia coli* (ETEC), *Staphylococcus aureus*, *Salmonella typhimurium, Listeria monocytogenes*, and *Aeromonas caviae* (Table 1), while other strains showed weak antibacterial activity against these pathogens. From the selected strains, HSM-1, HSM-10, and HSM-18 are *Lactococcus lactis* strains while HSM-14 is *Leuconostoc lactis* strain. Among these four isolates, HSM-14 showed the highest zone of inhibition: 12.5 ± 3.2, 8.2 ± 1.2, 7.9 ± 1.1, 7.7 ± 0.9, and 7.1 ± 1.1 mm against *A. caviae*, *L. monocytogenes*, EHEC, ETEC, and *S. aureus*, respectively. Meanwhile, the clear inhibition against *S. typhimurium* was showed by HSM-10 with a 7.9 ± 1.4 mm inhibition zone. Based on our results, *A. caviae* was recognized as the most sensitive indicator bacteria against the selected LAB species, while *S. aureus* and EHEC showed resistance.

**Table 1:** Antimicrobial activity of isolated lactic acid bacteria strains against major foodborne pathogens

Strain	*S. aureus*	EHEC	*S. typhimurium*	*L. monocytogenes*	ETEC	A. *caviae*
HSM-1	6.1 ± 0.8^b^	6.9 ± 1.3^a^	7.2 ± 1.8^a^	7.2 ± 1.8^ab^	6.6 ± 1.0^b^	7.4 ± 2.5^c^
HSM-10	6.0 ± 0.5^b^	7.1 ± 2.0^a^	7.9 ± 1.4^a^	7.8 ± 2.1^ac^	7.7 ± 1.7^ac^	10.2 ± 1.9^b^
HSM-14	7.1 ± 1.1^a^	7.9 ± 1.1^a^	7.8 ± 1.2^a^	8.2 ± 1.2^ac^	7.7 ± 0.9^a^	12.5 ± 3.2^a^
HSM-18	5.4 ± 0.5^b^	5.2 ± 0.6^b^	6.5 ± 0.5^a^	6.1 ± 1.9^b^	6.4 ± 1.1^b^	6.2 ± 0.7^c^

Note: Data are mean ± SD (*n* = 3). Mean values with different superscript letters (a–c) in the same row are significantly different, based on LSD test (*p* < 0.05)

LABs can produce antimicrobial substances, such as bacteriocins, organic acids, and proteins, which are capable of inhibiting the growth of pathogens [32]. Also, the antagonistic activity of LABs is related to the pH decrease of their growth environment [33]. In order to study the mechanism underlying the antagonistic activity, the fermentation broth of HSM-14 was treated using different methods before conducting the antibacterial assay. The results showed that the antagonistic activity totally disappeared when the pH value of the supernatant from fermentation broth changed into neutral, but the inhibition zone became visible for the substances obtained from fermentation broth under anaerobic condition (Fig. 1c). This suggested that the organic acids produced by the LABs under anaerobic condition should be probably responsible for the antagonistic activity.

### Hydrophobicity of the bacterial surface

The hydrophobicity assay showed that all the four selected strains had high hydrophobicity (Table 2). The hydrophobicity of HSM-1, HSM-10 and HSM-14 in n-dodecane (97.8 ± 0.6%, 97.9 ± 1.4% and 97.1 ± 0.7% respectively) and xylene (92.6 ± 1.2%, 94.6 ± 1.0% and 93.4 ± 1.5% respectively) which was higher than that in chloroform (82.5 ± 5.5%, 84.5 ± 4.0% and 75.0 ± 7.1% respectively), but the hydrophobicity of HSM-18 in chloroform (97.1 ± 0.7%) was higher than that in n-dodecane (86.8 ± 3.8%) and xylene (84.8 ± 1.9%). Therefore, we concluded that HSM-10 possessed the highest hydrophobicity.

**Table 2:** In vitro probiotic properties of isolated lactic acid bacteria strains

Strain	Auto-aggregation and co-aggregation with pathogens				Hydrophobicity			Heat-treatment		
	Auto-aggregation	*S. aureus*	*S. typhimurium*	*L. monocytogenes*	n-Dodecane	Chloroform	Xylene	50 °C	60 °C	70 °C
HSM-1	59.9 ± 4.9%^a^	52.0 ± 14.9%^a^	17.1 ± 6.3%^a^	25.2 ± 3.8%^ab^	97.8 ± 0.6%^a^	82.5 ± 5.5%^b^	92.6 ± 1.2%^a^	74.6%^a^	74.2%^a^	0.2%^b^
HSM-10	63.5 ± 13.1%^a^	35.7 ± 2.3%^a^	16.2 ± 4.2%^a^	37.6 ± 8.1%^ab^	97.9 ± 1.4%^a^	84.5 ± 4.0%^b^	94.6 ± 1.0%^a^	57.5%^a^	14.4%^b^	0.1%^c^
HSM-14	16.5 ± 4.1%^b^	51.1 ± 13.1%^a^	28.0 ± 3.0%^a^	38.1 ± 12.7%^a^	97.1 ± 0.7%^a^	75.0 ± 7.1%^b^	93.4 ± 1.5%^a^	32.7%^a^	11.6%^b^	0.4%^ac^
HSM-18	23.7 ± 2.3%^b^	52.1 ± 2.9%^a^	14.5 ± 4.7%^a^	22.7 ± 5.0%^b^	86.8 ± 3.8%^b^	97.1 ± 0.7%^a^	84.8 ± 1.9%^b^	9.0%^a^	0.0%^b^	0.0%^a^

Note: Data are mean ± SD (n = 3). Mean values with different superscript letters (a–c) in the same row are significantly different, based on LSD test (*p* < 0.05)

### Auto-aggregation and co-aggregation

As shown in Table 2, HSM-1 and HSM-10 showed the best auto-aggregation ability among the selected strains (59.9 ± 4.0% and 63.54 ± 10.7%, respectively). HSM-14 and HSM-18 exhibited low auto-aggregation ability comparatively (16.5 ± 3.4% and 23.7 ± 2.0%, respectively). Additionally, the all four strains showed high co-aggregation ability [34] to *S. aureus* (52.0 ± 14.9%, 35.7 ± 2.3%, 51.1 ± 13.1% and 52.1 ± 2.9%, respectively). For *L. monocytogenes*, high co-aggregation [34] was observed in HSM-10 and HSM-14 (37.6 ± 8.1% and 38.1 ± 10.3%, respectively), followed by HSM-1 and HSM-18 (25.2 ± 3.8% and 22.7 ± 5.0%, respectively). For *S. typhimurium*, only HSM-14 showed the highest polymerization ability (28.0 ± 3.0%).

### Heat treatment

HSM-1 showed the highest heat resistance, followed by HSM-10 and HSM-14 (Table 2). When LABs were treated at 50 °C for 5 min, the survival rate of HSM-10 (57.5%) and HSM-14 (32.7%) was obviously lower than HSM-1 (74.6%). However, HSM-18 had the lowest rate of heat resistance (9.0%). No significant differences were observed relative to the growth of HSM-1 at 60 °C and 50 °C (*p*-value 0.99). After heat treatment at 60 °C, 74.2% of HSM-1 population survived. However, the survival rate of HSM-10 and HSM-14 declined to 14.4 and 11.6% respectively. After heat treatment at 70 °C, the survival rate of strains dropped less than 1%. These results indicate that HSM-1 has better heat resistance as compared to the other three strains at 50 °C or 60 °C.

### Antibiotic susceptibility assay

The susceptibility of four LABs was also evaluated against 30 antibiotics of nine different classes, which were sorted in line accordance with their resistance profile to the tested drugs (Table 3) [35]. None of the strains tested were sensitive to all antibiotics [35]. HSM-1 was resistant to Amikacin, Tetracycline, Norfloxacin, and Sulfamethoxazole. HSM-10 exhibited resistance to 9 antibiotics and HSM-14 exhibited resistance to 10 antibiotics. HSM-18 was not sensitive to Kanamycin, Neomycin, Tetracycline, Doxycycline, and Sulfamethoxazole.

**Table 3:** The sensitivity of probiotic candidates against 30 antibiotics

Types of Drugs	Drug	μg/pill	HSM-1	HSM-10	HSM-14	HSM-18
Penicillins	Penicillin	10 U	39 (S)	39(S)	37(S)	38(S)
	Oxacillin	1	27 (S)	19(I)	20(I)	22(S)
	Carboxycillin	100	42 (S)	44(S)	33(S)	40(S)
	Piperacillin	100	48 (S)	29(S)	37(S)	42(S)
	Ampicillin	100	31 (S)	35(S)	31(S)	32(S)
Cephalosporins	Cephalexin	30	33 (S)	40(S)	29(S)	28(S)
	Cefazolin	30	47 (S)	25(S)	27(S)	45(S)
	Cefradine	30	34 (S)	44(S)	27(S)	32(S)
	Ceftazidime	30	28 (S)	44(S)	17(I)	29(S)
	Cefoperazone	75	41 (S)	40(S)	29(S)	37(S)
	Cefatriaxone	30	41 (S)	38(S)	26(S)	35(S)
	Cefuroxime	30	48 (S)	44(S)	36(S)	49(S)
Aminoglycosides	Amikacin	30	19 (I)	19(I)	11(R)	24(S)
	Kanamycin	30	25 (S)	21(S)	7(R)	20(I)
	Neomycin	30	22 (S)	17(I)	15(R)	20(I)
	Gentamicin	10	22 (S)	14(R)	12(R)	21(S)
Tetracycline	Tetracycline	30	14 (R)	13(R)	29(S)	14(R)
	Doxycycline	30	22 (S)	22(S)	28(S)	17(I)
	Minocycline	30	30 (S)	27(S)	35(S)	26(S)
Macrolides	Erythromycin	15	34 (S)	29(S)	28(S)	32(S)
	Midecamycin	30	34 (S)	29(S)	23(S)	26(S)
Glycopeptides	Vancomycin	30	31 (S)	25(S)	7(R)	26(S)
Quinolones	Norfloxacin	10	18 (I)	16(I)	29(S)	25(S)
	Ofloxacin	5	32 (S)	27(S)	29(S)	29(S)
	Ciprofloxacin	5	27 (S)	26(S)	22(S)	21(S)
	Furazolidone	300	23 (S)	17(I)	19(I)	29(S)
	Chloramphenicol	30	39 (S)	36(S)	36(S)	36(S)
	Clindamycin	2	41 (S)	37(S)	27(S)	40(S)
Sulfonamides	Sulfamethoxazole (SMZ/TMP)△	23.75/1.25	17 (I)	18(I)	8(R)	20(I)
Other classes	Polymyxin	300 IU	22 (S)	5(R)	7(R)	24(S)

Note: The zone of inhibition (diameter in mm) for each antibiotic was measured and expressed as susceptible, S (≥21 mm); intermediate, I (16–20 mm), and resistance, R(<=15 mm)

### Growth at different bile salts concentrations and low pH

**Fig. 2:**
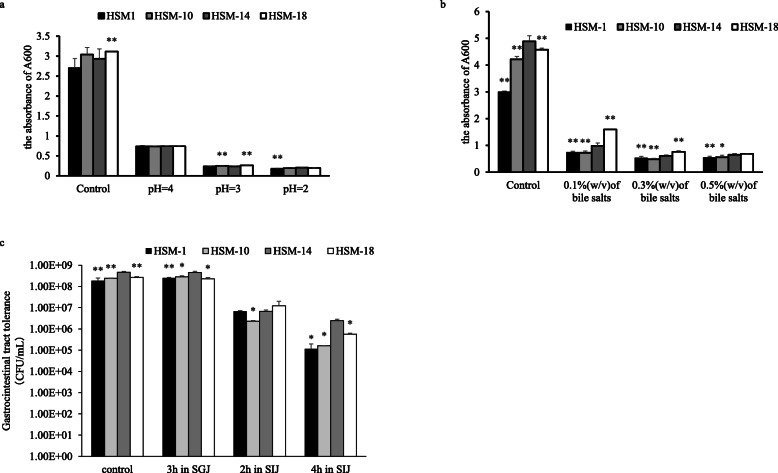
Microbial population of four LAB strains with a variable pH value and under bile salt conditions at 37 °C. ‘control’ in the figure refers to the experimental results corresponding to the control group (free of HCl, choline and SJC) in each experiment. **a** Absorsion of the isolates under acidic pH conditions for 24 h at 37 °C in MRS. **b** Absorsion of the isolates under bile salt conditions for 24 h at 37 °C in MRS. **c** Transit tolerance in simulated gastrointestinal conditions. Data shown are mean ΔSD of triplicate values of independent experiments

The selected LABs strains were incubated at 37 °C for 12 h in MRS at a variable pH range of 2.0, 3.0 and 4.0 to determine the acid tolerance (Fig. 2a). They showed certain viability under low pH. HSM-1, HSM-10, HSM-14 and HSM-18 grown best under the condition of MRS at pH 4.0 (27.33, 24.27, 25.22 and 23.75%, respectively). The survival rate ranged from 8 to 9% under the condition of MRS with adjusted pH of 3.0 and was 6–7% under the condition of MRS with adjusted pH of 2.0. To estimate bile tolerance, four strains were treated with 0.1, 0.3 and 0.5% of bovine choline and incubated at 37 °C for 12 h. All these four strains exhibited resistance to different concentrations of bovine choline. The bacterial viability decreased with the increase of bovine choline (Fig. 2b). In summary, HSM-1, HSM-10, HSM-14 and HSM-18 had the ability to grow and survive at low pH and in the presence of bovine bile, demonstrating that these strains could tolerate gastrointestinal environmental conditions.

### Resistance to simulated gastric and intestinal conditions

Survival of the four LABs after 3 h of SGJ treatment and 4 h of simulated pancreatic juice treatment were showed in Fig. 2c. These four LABs demonstrated the high survival rates after 3 h cultivation in the SGJ [36]. As expected, the results revealed that the survival rate of the four LABs decreased with the increase of treatment time. At pH 3.0, the survival rates of HSM-1 and HSM-10 increased by 0.125 log10 CFU/mL and 0.064 log10 CFU/mL, which showed their survival rates were higher than 100%, while the viability of HSM-14 and HSM-18 decreased by 0.012 Log_10_ CFU/mL and 0.065 Log_10_ CFU/mL. Besides, all four LABs have decreased their populations in the SIJ [37]. After 4 h exposure to the SIJ, HSM-1 decreased the most, about 3.208 Log_10_ CFU/mL, followed by HSM-10 decreased by 3.186 Log_10_ CFU/mL. HSM-14 and HSM-18 decreased by 2.28 Log_10_ CFU/mL and 2.674 Log_10_ CFU/mL respectively. HSM-1 was found to be most tolerant to bile salts that is about 2.28 Log_10_ CFU/mL. These four strains might become potential probiotic candidates. In view of the different survival rates, the smallest variation trend of HSM-14 indicated that it had the strongest survival ability and resistance in the simulated gastrointestinal environment.

## Discussion

In current study, we focused on the LABs with potential probiotic functions explored from Hu sheep milk. The increase in multidrug resistant organisms compromised the therapies of a growing number of infectious diseases for decades [38]. Thus, it is necessary and urgent to find new alternatives which are more effective against antibiotic resistant pathogens. In this study, different food borne pathogens for example *S. aureus*, ETEC, EHEC, *S. typhimurium*, *L. monocytogen,* and *A. caviae* were studied and had been found to be associated with certain gastrointestinal diseases including gastrointestinal infections [39]. Among the selected strains, HSM-14 showed the highest zones of inhibition against above mentioned pathogens.

Cellular hydrophobicity is the premise for probiotics to exert the beneficial effect of probiotics by eliminating intestinal pathogens, adhere to intestinal epithelial cells, and colonize the gastrointestinal tract [40]. In addition, the ability of cells to auto-aggregate makes a significant contribution of adherence to intestinal cells and avoids pathogens colonization. Co-aggregation ability of bacteria eliminates gastrointestinal pathogens by preventing them from adhering to host tissues. Potential probiotics should have the ability to colonize the intestinal tract and prevent the colonization of pathogenic bacteria [32]. HSM-1, HSM-10, HSM-14, and HSM-18 had high hydrophobic capacity. The co-aggregation scores of HSM-1, HSM-10, and HSM-18 with the pathogen *S. aureus* exceeded 50%, and the effect was better than *S. typhimurium* and *L. monocytogenes*.

Safety is also a significant consideration while selecting potential probiotics before they are available in the market for public usage [41, 42]. We evaluated the antibiotic resistance of LABs from Hu sheep milk. The results of antibiotics sensitivity showed that different antibiotics had different effects on the selected isolates. The risk of dissemination of resistant genes to other microorganisms is increasing, and potential probiotic strains should not have transferrable antibiotic resistance [1]. We will screen virulence genes and resistance genes in potential probiotics strains to further evaluate the safety of strains in the near future.

Survival and growth at low pH and in the presence of bovine bile are also desirable characteristics for potential probiotics strains. Human large intestine contains bile salt, whose concentration varies between 0.3% ~ 0.5%. Generally, the pH of gastric juice depends on the time of feeding, diet and it may vary from 1.5 to 4.5 [43, 44]. Growth and survival at low pH and in the presence of bovine bile are thought to be the most desirable characteristics for future probiotic strains. Probiotics need to be survival in the small intestine through acidic environment of the stomach and alkaline environment of the colon resisting bile salt. The pH of gastric juice is around 3.0, and the stomach gastric digestion can last for 3 h [14]. According to the previous study [45], the pH of 3.0, for 3 h was reported as the standard for screening probiotics for acid resistance. The physiological concentration of human bile ranges between 0.3 and 0.5%, the time for food to pass through the small intestine is about 4 h, and the average concentration of bile is 0.3%. By simulating the gastrointestinal environment and comparing the low pH environment with the high bile concentration environment, the feasibility of the experimental strain as a probiotic was also evaluated. The results indicated that HSM-1, HSM-10, HSM-14, and HSM-18 were resistant to low pH and high bile acid concentrations. Moreover, HSM-14 had the highest survival rate as compared to any of the other three strains in the experiments, and resistant to simulated gastric and intestinal conditions. The results showed that HSM-14 could be a promising probiotic candidate.

During heat drying processing of probiotics, a large number of probiotics bacteria dies because of their less thermal stability. During the pasteurization, milk sample should be under heat-treatment for a certain time. Pasteurization is a common method that used to eliminate the milk borne pathogens. It has shown that pasteurization can reduce the number of microorganisms in milk by about 20 times [46]. Holder pasteurization (62.5 °C, 30 min) is the most widespread method of milk processing at comparatively low temperature [47]. The typical treatment for pasteurization is the high temperature short duration method, where milk is heated to 72 °C for 15 s [46]. Therefore, in this heat resistance experiment, we chose a maximum temperature of 70 °C to explore the prospect of four LABs in industrial applications, including pelletizing and storage.

After the treatment at 70 °C, the survival rate of the isolates was less than 1%, and the heat resistance effect of HSM-18 was the worst. Adding certain substances can enhance the heat resistance of LABs, such as recombinant skim milk [48]. The heavy fat skimmed milk is rich in calcium, which can promote the growth of LABs and also improve the thermal stability of cell envelope protease.

These tests enabled a preliminary selection of strains having probiotic potentials, which can resist gastrointestinal conditions, and also have antibacterial effects on harmful bacteria. These trials are preliminary steps of a large number of assays devoted to select and characterize probiotic *Lactobacillus* strains as alternatives to antibiotics and aimed to decrease antibiotics usage to treat gastrointestinal diseases and stabilize the balance of intestinal microbial microbiota. We will screen certain virulence and resistance genes in potential probiotics strains to further evaluate the safety of strains in the future.

## Conclusions

In the present study, four LABs isolated from Hu sheep milk, HSM-1, HSM-10, HSM-14, and HSM-18, were found to have potential probiotic candidates’ properties. We evaluated these LAB strains through using a series of in vitro experiment for detecting probiotic candidate’s properties, which can be raised for the production of various kinds of food and pharmaceutical products. It was worth noting that HSM-14 had prominent advantages over other strains in terms of gastrointestinal tract tolerance and antimicrobial activities against pathogens, and it was believed to be the best candidate for potential LAB probiotics. It was suggested that the LABs procured might be used as bio-therapeutics against bacterial infection to humans. Our isolate is a potential candidate for application such as novel probiotic isolates which is used for human or animal food processing and drug production in the future. Nevertheless, further investigations are needed to evaluate the isolates in vivo and to assess the technical characteristics.

## Methods

### Screening and identification

According to instructions and consultants from experimental farm of Hu sheep (Yichang, China), the milk samples were collected aseptically and brought cooled to the laboratory. The Hu sheep milk samples were inoculated into MRS broth (OXOID Co., Ltd. Shanghai, China) and cultivated for 3 days at 37 °C under anaerobic conditions [49]. Identification of the isolates at genus level was carried out by using morphological and phenotypic methods. White and cocci shaped bacterial colonies were selected and further subjected to sub culturing on MRS agar (OXOID Co., Ltd. Shanghai, China) to obtain pure bacterial culture [50]. All isolates were stored at − 80 °C with 50% (v/v) glycerol and designated as HSM1 to HSM40. DNAs of the selected 40 strains were extracted and 16 s rRNA gene were amplified using the universal primers 27F (5′-AGAGTTTGATCMTGGCTCAG-3′) and 1492R (5′-GGTTACCTTGTTACG ACTT-3′) [51]. The PCR amplified products were sequenced by using Sanger sequencing (Sangon Biotech Ltd., China) and blast against NCBI nucleotide collection (nr/nt) database using BLASTN. The nearest match for each sequence was extracted, and taxonomy was assigned up to the species level. We counted the number of sequences assigned to unique species for subsequent analysis. The phylogenetic tree was constructed by using Maximum Parsimony (MP) model of software MEGA-X.

### Antimicrobial activity

Antimicrobial activity assays were determined using the agar well diffusion assay, as suggested by the previous study [52]. LABs were grown in MRS broth at 37 °C for 18 h, and pathogenic bacteria including *S. aureus* ATCC 25923, EHEC O157:H7 ATCC 43894, ETEC O149:K88 W25K [53], *L. monocytogenes* ATCC 19113, *S. typhimurium* ATCC 14028, and *A. caviae* ATCC 15468) were grown in LB broth (Sangon Biotech, Co., Ltd. Shanghai, China) at 37 °C for 12 h in an aerobic condition. Pathogenic bacteria were serially diluted with LB liquid medium up to 200 times and then flooded on LB agar plates and were kept at room temperature for 20 min for drying. Five wells were prepared in each agar plate by using a depth of 6 mm and a diameter of 5 mm sterile iron pipette. Precisely, 30 μL of LAB culture were added to each well, and one remaining well was filled with 30 μL of MRS broth as the negative control. These plates were then incubated in an aerobic condition at 37 °C for 24 h. After incubation, the zone of inhibition (ZOI) was measured by length meter, and the experiment was repeated at least three times.

### Hydrophobicity of the bacterial surface

The hydrophobicity of the bacterial surface was determined by the Microbial Adhesion to Hydrocarbon (MATH) assay [54]. Bacterial hydrophobicity was measured through using different organic solvents such as n-dodecane (Aladdin Co., Ltd. Shanghai, China), xylene (Sinopharm Chemical Reagent Co., Ltd. Shanghai, China), and chloroform (Sinopharm Chemical Reagent Co., Ltd. Shanghai, China). The LABs were grown in MRS broth at 37 °C for 24 h. Bacterial cell suspensions were then harvested by centrifugation at 6000 rpm for 10 min at 4 °C and washed twice with PBS (BBI Life Sciences Co., Ltd. Shanghai, China), which was resuspended in PBS (pH = 7.2) for an Optical Density (OD _600_ nm) of 0.8. One milliliter of n-dodecane, xylene or chloroform was added to 3 mL of bacterial cell suspension in a separate test tube, and vortexed for 120 s. The tubes were kept for 20 min at room temperature to separate the organic and aqueous phases. Later, the organic phase was removed, and the absorbance of the aqueous phases was determined at 600 nm, while PBS was used as a control. The experiment was repeated for three times, and the hydrophobicity percentage was determined as follows:

$$ \mathrm{Hydrophobicity}\ \left(\%\right)=\left[\left({\mathrm{A}}_0-{\mathrm{A}}_{\mathrm{t}}\right)/{\mathrm{A}}_0\right]\times 100\% $$

A_0_ represents the value of OD_600_ before mixing. A_t_ stands for the value of OD_600_ after mixing. The degree of bacterial hydrophobicity was classified as low (0–29%), medium (30–59%) or high (60–100%) [54].

### Auto-aggregation assay

Auto-aggregation assay was determined according to the previously reported method [55], with slight modifications. LABs were grown in MRS broth for 24 h at 37 °C. Then bacteria were harvested by centrifugation at 6000 rpm for 10 min at 4 °C. The harvested cells were then washed twice and resuspended in PBS (pH = 7.2) to achieve the OD_600_ of 0.8. Four milliliters of the bacterial suspensions were vortexed for 120 s and incubated at room temperature for 5 h. One milliliter of the above suspension was carefully taken up and the OD_600_ was determined. The experiment was repeated three times and auto-aggregation was calculated as follows:

$$ \mathrm{Auto}-\mathrm{aggregation}\ \left(\%\right)=1-{\mathrm{A}}_{\mathrm{t}}/{\mathrm{A}}_0\times 100\% $$

A_t_ represents the value of OD_600_ at time t = 5 h; A_0_ indicates the value of OD_600_ at t = 0 h.

### Co-aggregation assay

Co-aggregation assay was evaluated according to previously reported method [56]. Bacterial suspensions of LAB and pathogenic bacteria (*S. aureus* ATCC 25923, *S. typhimurium* ATCC 14028, and *L. monocytogenes* ATCC 19113) were prepared same as for auto-aggregation analysis. Equal volumes (2 mL) of each LAB and pathogenic bacteria cell suspensions were mixed and incubated at room temperature for 5 h without agitation. Control tubes containing 4 mL of single bacterial suspensions were also incubated under the same growth condition, and the OD_600_ was measured. The experiment was repeated for three times and the calculation formula for co-aggregation was addressed as follows:

$$ \mathrm{Co}-\mathrm{aggregation}\left(\%\right)=\left[\left({\mathrm{A}}_{\mathrm{x}}+{\mathrm{A}}_{\mathrm{y}}\right)/2-{\mathrm{A}}_{\left(\mathrm{x}+\mathrm{y}\right)}\right]/\left({\mathrm{A}}_{\mathrm{x}}+{\mathrm{A}}_{\mathrm{y}}\right)\times 100\% $$

Where A_x_ and A_y_ represent the absorbance of the two bacteria cell suspensions and A_(x + y)_ means the absorbance of mixed bacteria cell suspensions.

### Heat treatment

The LAB strains after 18 h cultivation in MRS broth were concentrated to 1.0 ml cell suspension and kept at the different temperatures of 37 °C, 50 °C, 60 °C, and 70 °C, respectively, for 5 min. The numbers of living cells were determined in terms of CFU·mL^− 1^. Each experiment was repeated three times to calculate the mean, the calculation formula for survival was as follows:

$$ \mathrm{Survival}\ \left(\%\right)=\mathrm{CFU}\cdotp {\mathrm{mL}}^{-1}\left(\mathrm{t}\right)\times 100/\mathrm{CFU}\cdotp {\mathrm{mL}}^{-1}\ \left({\mathrm{t}}_0\right) $$

t stands for 50 °C, 60 °C, and 70 °C while t_0_ representss for 37 °C.

### Antibiotic susceptibility assay

Antibiotic susceptibility assay of LAB isolates was performed on MRS agar via disc diffusion method [57]. At first, LAB isolates were grown in MRS broth at 37 °C to achieve the OD_600_ of 0.8. Then, LABs were added to the semi-solidified MRS medium and diluted at a ratio of 20,000 times. Next, they were shaked gently and poured into petri plate for solidification at room temperature. Antibiotics (Hangzhou Mirobial Reagent Co, Ltd) used in the test include penicillin (P, 10 U/disc), oxacillin (OX, 1 μg/disc), carboxycillin (CB, 100 μg/disc), piperacillin (PIP, 100 μg/disc), ampicillin (AM, 100 μg/disc), cephalexin (CA, 30 μg/disc), cefazolin (CZ, 30 μg/disc), cefradine (RAD, 30 μg/disc), ceftazidime (CAZ, 30 μg/disc), cefoperazone (CFP, 75 μg/disc), amikacin (AK, 30 μg/disc), kanamycin (K, 30 μg/disc), neomycin (N, 30 μg/disc), gentamicin (GM, 10 μg/disc), tetracycline (TE, 30 μg/disc), doxycycline (DX, 30 μg/disc), minocycline (MI, 30 μg/disc), erythromycin (E,15 μg/disc), midercamycin (MD, 30 μg/disc), vancomycin (VA, 30 μg/disc), norfloxacin (NOR, 10 μg/disc), ofloxacin (OFX, 5 μg/disc), ciprofloxacin (CIP, 5 μg/disc), furazolidone (FZ, 300 μg/disc), chloramphenicol (C, 30 μg/disc), clindamycin (CC, 2 μg/disc), cefatriaxone (CTR, 30 μg/disc), cefuroxime (CXM, 30 μg/disc), sulfamethoxazole (SXT,23.75/1.25 μg/disc), and polymyxin (PB, 300 IU/disc). At last, antibiotic discs were placed on medium surface and then LAB strains were incubated anaerobically at 37 °C for 24 to 48 h. After incubation, diameter of transparent zones was measured and to make the experiment statistically significant it was repeated three times. The results were compared with the values designated by the Clinical and Laboratory Standards Institute.

### Growth at different bile salts test and growth at low pH

The tolerance ability of the strains to bile salt and low pH environment were conducted following Ramila Azat method [58]. Cells of the selected strains were grown in MRS broth for 12 h at 37 °C. The freshly grown culture of strain (4%, v/v) was inoculated in MRS broth containing 0, 0.1, 0.3, and 0.5% (w/v) of bile salts under anaerobic conditions (Huayuehang Instrument Co., Ltd. Guangdong Province, China) for 12 h at 37 °C. The method used to evaluate the proliferation of the strains at low pH was similar to the above protocol. Cells of the selected strains were grown in MRS broths with variable pH values of 2.0, 3.0, and 4.0. The different pH solutions were adjusted by adding 1 M HCl (Sangon Biotech Co., Ltd. Shanghai, China) under anaerobic conditions for 12 h at 37 °C. The OD_600_ was measured and calculated in comparison with the control group (choline free, HCl free). The experiment calculation formula was as follows:

$$ \mathrm{Survival}\ \left(\%\right)={\mathrm{OD}}_{600}\left(\mathrm{experiment}\right)\times 100\%/{\mathrm{OD}}_{600}\left(\mathrm{control}\right) $$

### Resistance to simulated gastric and intestinal conditions

In order to determine the tolerance of LABs in the simulated gastric and intestinal conditions, the adaptation of the four LAB strains were assessed in vitro according to the reported method [59]. In short, simulated gastric juice (SGJ) was made by dissolving 3.0 g pepsin (BBI Life Sciences Co., Ltd. Shanghai, China) (from Porcine Stomach BC Grade) in one liter sterile saline solution (0.9%, w/v), and the pH was adjusted to 3.0 by using sterile 1 M HCl. Simulated intestinal juice (SIJ) was formulated by the addition of 0.3 g/L bile salts (Sigma, USA) and 1.0 g/L trypsin (Sinopharm Chemical Reagent Co., Ltd. Shanghai Province, China) in sterile saline solution (0.9%, w/v), and the pH was adjusted to 3.0 by using sterile 1 M NaOH (Sangon Biotech Co., Ltd. Shanghai, China). The prepared solution was filtered using 0.45 nm filter membrane (Sangon Biotech Co., Ltd. Shanghai, China). LABs were continuously activated for 18 h in MRS broth at 37 °C. One milliliter of bacterial cell suspension was harvested by centrifugation at 3000×g for 10 min at 4 °C and dissolved in MRS broth (500 μl) and SGJ (500 μl) miscible liquids, and incubated at 37 °C for 2 h at 300 rpm and then the bacteria were resuspended in SIJ and incubated in the same condition for 4 h. The cell suspensions were incubated at 37 °C for 0, 3, 5, and 7 h and then cultivated on the MRS agar for 24 h. Then, the number of viable cells was estimated. Log_10_ CFU/mL reduction was determined by the difference of 0, 3, 5, and 7 h Log_10_ CFU/mL.

### Statistical analysis

All the results (except heat-treatment) were presented as the mean value and standard deviation of three replicates. Statistical analysis was performed using SPSS software (v22.0 for Windows; IBM Corp.). For antimicrobial activity, the data was subjected to a LSD (L) test, the mean value was separated using Duncan’s multiple-range test, and statistical significance was defined as *p* < 0.05.

## Data Availability

Data sharing is not applicable to this article as no datasets were generated or analysed during the current study.

## Abbreviations

16SrRNA: 16S ribosomal ribonucleic acid
CFU/ml: Colony forming unit per millilitre
LAB: Lactic acid bacteria
PCR: Polymerase chain reaction
OD: Optical density
DNA: Deoxyribonucleic
EHEC: Enterohemorrhagic *E. coli* O157:H7 ATCC 43894
ETEC: Enterotoxigenic *Escherichia coli* O149:K88 W25K
*S. aureus*: *Staphylococcus aureus*
*S. typhimurium*: *Salmonella typhimurium*
*L. monocytogenes*: *Listeria monocytogenes*
*A. caviae*: *Aeromonas caviae*
MRS: De Man, Rogosa an Medium (Modified MRS Medium Base)
LB: Luria-Bertani
SGJ: Simulated gastric juice
SGJ: Simulated gastric juice
Log_10_ CFU/mL: Denary logarithm of Colony forming unit per millilitre
